# Kidney Exchange Program Reporting Standards: Evidence-Based Consensus From Europe

**DOI:** 10.3389/fpubh.2021.623966

**Published:** 2021-02-11

**Authors:** Bart Smeulders, Michal A. Mankowski, Joris van de Klundert

**Affiliations:** ^1^Department of Mathematics and Computer Science, Eindhoven University of Technology, Eindhoven, Netherlands; ^2^Computer, Electrical, and Mathematical Sciences and Engineering Division, King Abdullah University of Science and Technology, Thuwal, Saudi Arabia; ^3^Prince Mohammad Bin Salman College, King Abdullah Economic City, Saudi Arabia

**Keywords:** kidney exchange, Living donation, reporting standard, end stage renal disease (ESRD), kidney exchange program

## Abstract

**Background:** Kidney Exchange Programs can play an important role to increase access to the life saving and most cost-effective treatment for End Stage Renal Disease. The rise of national KEPs in Europe brings a need for standardized performance reporting to facilitate the development of an international evidence base on program practices.

**Methods:** We systematically searched and reviewed the literature to extract kidney exchange program performance measures. Reported measures were initially categorized as structure, process, and outcome measures. Expert feedback was used to redefine categories and extend the set of measures to be considered. Using the Delphi method and a panel of 10 experts, the resulting measures were subsequently classified as mandatory (Base set), optional (Extended set), or deleted.

**Results:** Out of the initial 1,668 articles identified by systematic literature search, 21 European publications on kidney exchange programs were included to collect performance measures, accompanied by three national program reports. The final measurement categories were Context, Population, Enrollment, Matching, Transplantation, and Outcomes. The set of performance measures resulting from the literature review was modified and classified as mandatory or optional. The resulting Base set and Extended set form the kidney exchange program reporting standard.

**Conclusions:** The evidence-based and consensus-based kidney exchange program reporting standard can harmonize practical and scientific reporting on kidney exchange programs, thus facilitating the advancement of national programs. In addition, the kidney exchange program reporting standard can promote and align cross-national programs.

## Introduction

With a mortality number of 1.2 million, Chronic Kidney Disease (CKD) is the 11th most common cause of death globally, and ranks 13th in Europe (1). It is a progressive disease of which End Stage Renal Disease (ESRD) is the last stage. The relative contribution of ESRD to European mortality is increasing, and currently stands at 1.58% in Europe, while CKD accounts for 1.06% of the total burden of disease in Europe (1). The default treatment for ESRD is dialysis (2). Dialysis incurs higher costs and lower quality of life than transplantation (2). In many European countries, transplantation programs have emanated from deceased donor programs. They have been complemented by living donor programs to promote access and quality of transplantation (3, 4). Live donation traditionally has been restricted to family members or close friends of a patient donating one of their two kidneys. Unfortunately, even when a living donor is available, transplantation may not be feasible because of incompatibility between the patient and the donor.

Over the past two decades, Kidney Exchange Programs (KEPs) have emerged in many countries to promote the benefits of living donor kidney donation. They particularly service pairs consisting of a patient and a living donor willing to donate to the patient for whom transplant is not feasible because the donor is not compatible with the patient. The KEPs “exchange” donors among patients so that patients are matched with compatible donors after which corresponding transplants take place.

Across the world, the design and developments of KEPs have varied considerably. The variations often are solutions to resolve country specific challenges such as small population size (Iceland) geography (Australia) and pre-existing deceased donor programs (Spain) (3, 4). In addition, the variations have arisen from challenges posed by differences in legislation. For example, countries such as Finland and Germany legally forbid living donation to recipients with whom the donor doesn't have a close relationship, whereas in other countries, such as France and Switzerland, altruistic donation is not legally allowed (3, 4).

Another main difference among KEPs arises from differences in national governance models. In many countries, national governance is limited to providing regulation for KEPs. The regulatory frameworks then may govern single center KEPS, such as in the Czech Republic and Slovakia, or KEPs between a small number of centers, such as in Poland. In the USA, less than half of the 250 living donor transplant centers participate in the nationwide KEP administered by UNOS, the organization which manages the nation's organ transplant system under contract with the federal government. In addition, the National Kidney Registry and the Alliance for Paired Donation operate nationwide and regional and single-center KEPs exist. At the other end of the governance spectrum, a national KEP has naturally emerged in the UK with its nationwide public health system. We refer to (3, 5) for a more detailed discussion of KEPS in Europe and across the globe, including in Australia, Canada and South Korea.

The dynamics of the emergent KEPs and their contextual differences have not only resulted in a variety of KEPs but have also brought along a variety of KEP performance measures reported. While this variety of reporting practices promotes novel approaches and viewpoints, it hampers comparability of practices and performance, and ultimately the development of an evidence base on KEP performance, as is beneficial for existing and newly emerging KEPs and most importantly for patients suffering from ESRD. Our research aims to synthesize reported measures and develop a consensus-based set of reporting standards.

Our research focusses on reporting for national KEPs. Moreover, we limit the scope to European KEPs. The reasons for limiting the scope to nationally coordinated KEPs in Europe are 2-fold. First, it serves to limit contextual differences, which complicate consensus and harmonization of standards. European countries and health systems differ essentially from other countries reporting on KEPS, such as Australia, China, India, Iran, Japan, Korea, and the USA, which translate to differences in KEPS and in KEP reporting priorities and practices. Second, Europe is presently witnessing various initiatives for cross-national KEPs (6), which especially call for harmonization of performance measures and reporting among European KEPs. These initiatives include bilateral KEPs between two countries, such as the Czech-Austrian kidney exchange (6) or KEPs involving multiple countries such as the Scandia Transplant Kidney Exchange Program (3).

To accomplish our research aim, we address two research questions. First, we seek to comprehensively map present scientific and practical reporting by European national KEPs. On this basis, we then proceed to develop unified reporting standards for European KEPs. The resulting Kidney Exchange Program Reporting Standards (KEPREPS) consist of a Base Set of essential, mandatory measures and an Extended set of optional measures, valuable to report for some programs. The presented work is developed within the EU COST Action 15210 entitled European Network for Collaboration on Kidney Exchange Programs (ENCKEP).

## Methods

The method section contains two main parts. First is a systematic review and synthesis of European literature on Kidney Exchange Programs. The results of this literature review served as the starting point for the second stage of developing a reporting standard. In this stage, we solicited and processed several rounds of expert feedback following the Delphi method to derive consensus on the Kidney Exchange Program Reporting Standards (KEPREPS) reporting standard. The details of both methods are specified below.

### Systematic Literature Review

Data on KEP performance measures were extracted from peer-reviewed English language scientific journals and conference proceedings and (annual) reports from Europe's three largest kidney exchange programs: The Netherlands, Spain, and The United Kingdom. A publication was considered 'European' if it reported data from a European KEP or if the first author was affiliated with a European institution.

After extensive consultation with an expert librarian, we included articles from Embase, Medline, Web of Science, and Cochrane matching the following query:

[(kidney OR donor^*^ OR transplant^*^ OR graft^*^) AND (exchange^*^OR pair^*^-exchange^*^ OR pair^*^-donation^*^ *OR sharing)]*

OR

[(pair^*^-exchange^*^ OR pair^*^-donation^*^ OR ((exchange^*^OR sharing OR chain) AND (donor^*^ OR donation^*^ OR kidney^*^))] AND (renal^*^ OR kidney^*^)]

In the first round, the first and third author screened the title and abstract of all articles and excluded the articles for which both agreed on exclusion. In a second round, the same two authors considered the full texts of the non-excluded articles, again deciding to exclude only if both authors agreed on exclusion. Differences in assessment on exclusion were resolved by consensus.

Next, all included articles were screened by the same two authors to collect all performance measures reported. Again, the two authors ensured full consensus on each of the reported measures for each of the included articles. Lastly, the first author included all performance measures explicitly included in the annual reports of the British, Dutch, and Spanish KEPs.

#### Measure Categories

The measures extracted from the literature were subsequently categorized from two perspectives. Firstly, we distinguished different types of measures, using Donabedian's seminal Structure-Process-Outcome framework (7). This resulted in three initial categories of KEP measures-structure measures, process measures, and outcome measures. A fourth set of measures on the population participating in the KEP was added to ensure all measures were categorized. The second perspective regarded the frequency of reporting, which we interpreted to indicate the relevance of the measures. Hence, based on the reporting frequency, we provided an initial classification of measures. Commonly reported measures were initially classified as mandatory, less commonly, but still regularly reported measures were initially classified as optional, and the remainder as exceptional.

#### Base Set and Extended Set

The systematic review results were presented to the large group of ENCKEP participants, representing 28 predominantly European countries. This large audience of representatives provided feedback and proposed additional measures.

Based on this initial expert feedback, the categories were redefined and formed the input for a procedure following the Delphi method with a set of 10 volunteering expert participants of ENCKEP (8). The experts in the Delphi panel responded to two questionnaire rounds. During the first questionnaire round, the experts categorized measures into three groups: Base set, Extended set, and Other. The Base Set consisted of measures that should be reported by every KEP. The role of the Extended set was to incorporate important but non-essential measures, while the category “Other” accommodated non-essential measures to be excluded from the standard.

The experts were also given the opportunity to provide motivation for their answers and make additional comments. Based on the results of the first round, we calculated the average score for each measure granting 3/2/1 points for Base Set/Extended Set/Other and rounded the score for each measure to the nearest integer. This rounded score was then converted into an initial classification of each measure into Base set (score ≥ 2.5), Extended set (2.5 ≥ score ≥ 1.5), or Other (score <1.5).

In the second round, we presented the resulting Base set and Extended set and asked each expert to agree or disagree. The experts were requested to motivate any disagreement to facilitate further improvement or finalize decision making on the classification. A third round would have been conducted in case of a lack of consensus but was not necessary. The resulting classification formed the targeted reporting standard KEPREPS.

## Results: Systematic Literature Review

The systematic literature search yielded 1,668 articles. After scanning of titles and abstracts, 346 were included. After reading of the full text (thus eliminating conference papers and posters), 116 articles remained, 21 of which were regarded as European. To this set, we added three publicly available annual reports of major European KEP programs.

The review distinguishes observational empirical studies and (simulation) model studies. No controlled experimental studies have been reported. There are 16 observational studies, including three recent annual reports from the aforementioned KEPs, and 8 model studies. With one exception (9), the observational studies are quantitative studies. The (simulation) model studies used real-life data, generated data, or a combination of both. Table 1 categorizes the included publications.

**Table 1:** List of included European publications and their categorization.

	**Publication type**	**References**
Observational	KEP reports	(10–12),
	Journal articles	(13–25)
Model	Real data	(9, 26–29)
	Simulated data	(30–32)

The majority of the included studies originate in the Netherlands (13/24) and consider the long-running Dutch KEP. By contrast, only 2 UK documents are included, despite the large size of the UK program. Seven publications are from 2005 to 2009, 9 from 2010 to 2014, the remaining eight from 2015 onwards. Detailed information per included article, including the reported measures, is available as Supplementary Material.

### Population Measures

Population measures are the measures describing the donors and patients involved in the kidney exchange program. This data can be further divided into three main categories, information regarding program size, demographic data, and medical data.

### Program Size

Program size measures are commonly reported. For instance, 12 out of 16 observational studies report the number of patients participating in the KEP, as do seven out of eight model studies. Of the model studies, five publications report on computational challenges in relation to program size. Papers not reporting on program size typically have a specific, different focus, such as validation of virtual crossmatch procedures (9) or KEP transplant outcomes compared to living-related transplant outcomes (13).

### Demographics

Demographic data is commonly reported in the observational literature. Age (10/16), the relationship between donor and patient (8/16) and gender (5/16) information is provided often, mainly in papers describing functioning KEP programs. Ethnicity is only mentioned once (14).

In the simulation literature, demographic data is nearly absent. Within this literature, population data is focused on characteristics with a direct impact on the kidney exchange graph.

### Clinical Population Measures

The composition of the KEP pool with regards to ABO and immunological characteristics can have a large impact on the overall and individual outcomes. These measures also shed light on patient enrolment causes.

In the observational literature, the type of incompatibility (ABOi, positive crossmatch) within a pair is reported in 11 out of 16 papers. ABO information for donors or candidates (7/16) and patient PRA (8/16) are also commonly reported. In some cases, this information is given by the type of incompatibility. ABO information for patients is often limited to the number of blood type O patients. Reporting on incompatibility types is often in combination with subsequent reporting on outcomes, e.g., transplant probability per incompatibility type (see below).

Simulation papers have less commonly reported on the ABO and PRA typing (3/8) and type of incompatibility (2/8) explicitly. However, an additional two papers refer to the data simulator they employ, which addressed these measures too. Table 2 summarizes the reporting of population measures.

**Table 2:** Reporting of, population, structural, process, and reporting measures by type of study.

		**Observational**	**Simulation**
**Population measures**			
	Number of documents	16	8
Program size	# Patients or pairs	12	8
	# Non-directed donors	2	0
Demographics	Gender	6	1
	Age	11	2
	Ethnicity	1	0
	Pair relationship	9	0
Medical	PRA level	9	4
	ABO	8	4
	Pair incompatibility	11	3
**Structural and process measures**			
	Number of documents	16	8
KEP structure	Number of centers	5	0
	Enrolment rate	4	1
	Pairs per run	6	2
Match before crossmatch	Identified cycles (by length)	6	4
	Length of longest cycle/chain	2	3
	Patients matched	5	7
Process outcome	Transplants canceled due to crossmatch	4	2
	Transplants proceeding	13	5
	Transplants proceeding by incompatibility	6	1
	Transplants proceeding by ABO	6	2
	Average time to match	3	3
	Patients remaining in pool	6	1
	Abandonment rate	6	1
	Transplants outside KEP	7	0
Others	Cold ischemia time	2	0
	Computation time	0	3
	Transplant increase over other programs	2	0
**Reporting measures**			
	Number of documents	16	8
Transplant outcomes	Graft survival	5	0
	Patient survival	4	0
	Acute rejection rate	3	0
Descriptive statistics matched patients	PRA	5	0
	Age and gender	3	0
	Patient ABO	3	0
	Relationship to donor	2	0
	Incompatibility to donor	1	0
	Ethnicity	1	0
Descriptive statistics unmatched patients	PRA	3	0
	Age and gender	2	0
	Patient ABO	2	0
	Relationship to donor	2	0
Descriptive statistics matched donor	ABO	2	0

### Structure Measures

There is little reporting on structural characteristics. Five (observational) studies report on the number of transplant centers involved. Four studies report on the spread of transplants over the transplant centers, three of which are model studies.

### Process Measures

As depicted in Table 2, five studies report on enrolment rates, only one of which is a model study. For the matching process, ten studies report on cycle lengths and numbers before HLA crossmatching, six of which are observational. This topic may have received more interest from model studies because cycle length received considerable attention in the scientific literature in relation to computational complexity. Likewise, three of the five studies which report the length of the longest cycle and/or chain are model studies.

The process (outcome) measure receiving the most attention is the number of transplants. As much as 13 of the 18 observational studies and five out of eight model studies report the total number of transplants proceeding. Seven of these studies distinguished ABO incompatible and crossmatch incompatible pairs, one of which was a model study. Six studies reported transplants per blood type. Two studies reported on the number of blood type identical transplants, and one study reported on ABO incompatible transplants.

A closely related process measure is the number of matched patients before crossmatch. This measure is reported by 12 studies and by all but one of the model studies. A next closely related measure is the number of transplants canceled because of negative crossmatch. This measure is reported by six studies, only two of which are model studies.

Eight studies (of which six observational) report the average number of patients included per match run, and six studies (of which three are observational) report the average time until being matched. This average can be the overall average but may also distinguish blood types and highly sensitized patients. Seven studies report on the number of pairs remaining in the pool, of which six are observational. The same set of seven studies also reported on the abandonment rate. Seven observational studies reported on the number of patients who received a transplant outside of the exchange program. Several other less frequently reported measures are provided in Table 2.

### Outcome Measures

In comparison to process or population measures, outcome measures have received less attention. The most frequently reported outcome measures are graft survival rate (five observational studies), patient survival rate (four observational studies), and acute rejection rates (three observational studies). The only qualitative study included reported on psychological outcomes, such as psychological distress and complaints, and the need for support.

Five observational studies-four of which are Dutch-report on descriptive statistics for matched and unmatched patients. All these studies report on PRA levels of matched patients and four for unmatched patients. The age and gender of donors and recipients (matched patients), as well as recipient ABO type, are reported by three studies. Less frequently reported outcome measures can be found in Table 2.

## Results: Toward a Reporting Standard

The above results were discussed with a broad expert panel of ENCKEP participants who proposed additional measures. The discussion led to a revised categorization. The three resulting main categories are: Context Information, Process Measures, and Outcome Measures. The Context Information is subdivided into measures on the program, on participating individuals (recipients and donors) and on pairs. The process measures are partitioned into three sequential subcategories: Enrolment, Matching, and Transplantation.

For each of the categories, measures were classified as essential (Base Set), important but non-essential (Extended Set), or not important (Other) by a Delphi panel of 10 experts from France, Hungary, Italy, The Netherlands, Portugal, Spain, Switzerland, and United Kingdom. In a second Delphi round with the same panel (except one expert), the averaged classifications (see methods section) were proposed to the panel for approval.

The resulting classification, as presented in Tables 3, 4, was approved nearly unanimously. We refer to the Appendix for a total of 16 exceptions and expert reservations. Sometimes, disagreement or reservation was because of legal considerations (collecting data on ethnicity is not allowed) or regulatory conventions (reporting measures for the living donor program as a whole). Five of the measures involved were from the Base Set. In each of these cases, one expert disagreed. For varying reasons, the corresponding items were kept in the Base Set. For the items in the Extended Set, we judged minor disagreement was not problematic as reporting of items in the Extended Set is optional anyway.

**Table 3:** Base set of context information, donor/recipient/pair attributes, and performance measures suggested to report by every KEP.

**Measure name**	**Definition**	**Proposed format**
**Context information**		
Definition incompatibility	Complete (in)compatibility definition used in the KEP	Narrative
Acceptable mismatch possible in KEP	Program for patients qualifying for a 'mismatched donor (cd. eurotransplant acceptable mismatch program)	Narrative
**Recipient and donor attributes**		
Blood Type	Recipient and donor blood type	Distribution
cPRA	Recipient calculated panel reactive antibodies	Average value/distribution
Prior Transplants	Recipient prior transplants	Number/percentage with prior transplant
Gender	Recipient and donor age	Distribution/average
Age	Recipient and donor gender	Total/percentage per gender
Type of incompatibility	Pair's type of incompatibility (ABO, positive crossmatch)	Percentage among categories/narrative
Relationship	Relationship between donor and incompatible recipient	Percentage among categories/narrative
**Enrolment performance measures**		
Number of pairs	Number of pairs registered in the KEP	(Total/average) over the reporting period
Number of non-directed donors	Number of non-directed donors registered in the KEP	(Total/average) over the reporting period
**Matching performance measures**		
Pairs matched	Total number of matched pairs	(Total/average) over the reporting period
Pairs left unmatched	Total number of unmatched pairs	Total/average over the reporting period (e.g., remaining at the end of the reporting period)
Identified cycles/chains (by length)	Cycles and chains found in the matching, by number of patients in the cycle/chain	Total/average over the reporting period, by cycle/chain length.
Number of match failures	Total number of matches found that have failed	Total/average number across the reporting period
Reasons for match failures	Synthesis on match failures reasons	Narrative including common causes
HLA matching	Degree of mismatch in donor and candidate HLA profile	Average value (0–6)/minimium, average, maximum/distribution
Age matching	Age gap between donor and recipient	Average value/minimium, average, maximum/distribution
**Transplant performance measures**		
Pairs transplanted	Number of pairs transplanted	Total average number across the reporting period
Pairs not transplanted	Number of pairs not transplanted since enrolled	Total/average number across the reporting period (e.g., remaining at the end of period)
Pairs transplanted outside of KEP	Number of pairs enrolled in the KEP but transplanted outside the program	Total number across the reporting period
Average time to transplant	Time from the enrolment to transplant	Average value over the period
Non-directed donor utilization	Number of donations from non-directed donors	Total/average number across the reporting period, ratio
Increase in the total number of transplants due to KEP	Increase in the total number of kidney transplants due to KEP (all living and deceased donation)	Total/average number across the reporting period/rate
Percentage of KEP transplants as part of the total living donor program	Percentage of KEP transplants as part of the total living donor program	(Average) Percentage across the reporting period
**Outcome measures**		
Patient Survival	Living donor transplant recipients survivors as a percentage of KEP transplant recipients	(Average) Percentage
Donor Survival	Living donors surviving as a percentage of KEP transplant donors	(Average) Percentage
Graft Survival	Living donor transplant grafts surviving as a percentage of KEP transplanted grafts	(Average) Percentage

**Table 4:** Extended Set of context information, donor/recipient/pair attributes, and performance measures to report by KEPs.

**Measure name**	**Definition**	**Proposed format**
**Context information**		
MFI–threshold for (in)compatibility	Mean fluorescence intensity (MFI) level thresholds defining (in)compatibility criteria	Narrative
Matching algorithm	Matching algorithms used in the KEP	Description of algorithmic principles
Prevalence of need for a kidney transplant	National/regional population incidence and prevalence of ESRD	Incidence and prevalence numbers
Donor or organs travel	Distance that donor or organ traveled for transplant	Narrative
Definition cPRA	Exact calculation definition of cPRA	Descriptive
Other programs–where is the KEP in the system of other transplant programs.	Environment of other KEPs, living and deceased donation programs	Narrative
Outcomes of alternate transplant programs	Outcome measures of competing KEPs, living and deceased donation programs	Descriptive
**Recipient and donor attributes**		
Nationality	Recipient and donor nationalities	Number/percentage per country
Ethnicity	Recipient and donor ethnicity	Number/percentage ethnicity
Social demographics	Recipient and donor socio-economic information expressed statistically, also including employment, education, income	Distribution/rate
**Recipient attributes**		
Match probability	Recipient match probability within a KEP	Average value/distribution
**Donor attributes**		
LKDPI	Donor living donor kidney transplantation index	Average value distribution
**Matching performance measures**		
Computation time	Run-time of algorithms required to output matching pairs	Average value
**Transplant performance measures**		
Cold Ischemia time	CIT for kidneys transplanted in the program	Average value/minimum, average, maximum/distribution
Number of organs recovered from failed KEP matches	Organs initially recovered for a KEP matched transplant but used otherwise because of transplant cancellation	(total/average) over the reporting period
**Outcome measures**		
Number of rejection episodes	Number of rejection episodes among transplants within the KEP	Total/average number across the reporting period
Number of acute rejections	Number of acute rejections among transplants within the KEP	Total/average number across the reporting period
QALY for recipients	Quality-adjusted life years for recipients since transplant	Expected Total/average over multiple year horizon
QALY for donors	Quality-adjusted life years for donors since transplant	Expected total/average over multiple year horizon
Cost measures	Cost of transplantations including KEP maintenance costs	Total cost/cost per transplant across the reporting period

Table 3 shows the resulting Base Set. Among the added context measures is the definition of incompatibility, considered as necessary context information to interpret reporting on other measures. From the European perspective, participant participation in the Eurotransplant Acceptable Mismatch Program (33) (or similar program for highly sensitized candidates) was also considered essential. For recipient and donor attributes, the Base Set contained blood type, gender, cPRA, and age. Experts also judged reporting on relationship and type of incompatibility for enrolled pairs to be mandatory.

The Base Set process and outcome measures are presented in Table 3 Experts classified all proposed matching measures as essential (Base Set) except for computation time. In addition to the measures from the review, measures on KEP transplants as a percentage of the total living donor program, and the total increase in donation caused by the KEP are included in the Base Set. While not frequently reported in the systematic review, outcome performance measures on patient, donor, and graft survival were also selected as Base Set measures.

### Toward a Guideline on Reporting: Extended Set

Table 4 shows all context information, process, and outcome measures considered to be important but not essential and, therefore, to be included in the Extended Set. Most of the context information, including MFI thresholds, matching algorithm, organ/donor travel, and cPRA definitions, ended up in the Extended Set. The same goes for nationality, ethnicity, social demographics, donor's LKDPI (34), and recipient's match probability. Many of these measures were not in the systematic literature review results. The Extended Set also contained additional outcome measures relating to quality-adjusted life years (QALYs) for donors/recipients, number of rejections, and cost measures.

## Discussion

The results presented above provide a Reporting Standard for European KEPs based on systematic literature review and expert opinion collected from a panel of practitioners and scientists from a variety of European countries and KEPs. The literature review made clear that not all existing European KEPs have reported on performance in the scientific literature. Moreover, it is remarkable that more than half of the publications are from The Netherlands, as the UK and Spain have larger KEPs but only modestly contributed to the European evidence base in the form of peer-reviewed scientific publications.

Living donor transplantation, as promoted by KEPs, is evidenced to be the most cost-effective treatment for End Stage Renal Disease (35–37). Given the importance of cost-effectiveness in current health policy and decision making, the lack of KEP reporting on cost measures is remarkable, as is the fact that outcome measures (effects) are among the least reported. However, measures on cost and effects (outcomes) have been included in the Base Set of KEPREPS upon expert suggestion and approval. Measures of equity and fairness, e.g., in relation to PRA level, blood type, and ethnicity, have received little attention in the European literature and are not included.

The main and intuitive proxy for outcomes in KEPs has typically been the process indicator number of transplants, which is indeed the most reported measure. It signals that much of the reporting and assessment of KEPs has focused on the process rather than on the outcomes. This may be due to the need to focus on mastering and improving the operations of KEPs in the initial years of developing KEPs. It may alternatively be explained by the view that the relation between processes and outcomes are well-understood, and hence the outcome performance can be measured and managed by the process measures, such as the number of transplants. In any case, the process measures-subdivided into enrolment, matching, and transplantation measures-form the largest set of measures in KEPREPS and are pre-dominantly included in the Base Set.

The process and outcome measures require information on the context to be appreciated. Factors such as age, sensitization, blood type distribution, et cetera, are important to interpret process and outcome performance. Hence, the experts have considerably expanded the collection of measures providing context information, compared to the measures found by systematic literature review. Most of these measures are seen as important but not essential and are included in the Extended Set.

The Base set and Extended set of KEPREPS together facilitate unified scientific and practical reporting on KEPs. Moreover, KEPREPS serves to enhance the practical relevance of model studies, which so far have differed quite substantially in their reporting, hampering their usefulness to inform and improve practice. In view of the importance presently attached to (health) outcomes and the difficulties faced globally to sustainably finance health systems, we believe that the inclusion of costs and outcomes in KEPREPS are especially valuable. Cost and health outcome measures have been hardly reported in scientific literature thus far. Hence the evidence base for the cost-effectiveness of KEPs is lacking, while a sound evidence base provides legitimacy to policy advancements of KEPS and especially larger coordinated KEPS which appear to be more effective than single center KEPS (23). Adoption of the KEPREPS standards by researchers and policy makers can therefore contribute to reducing the burden of ESRD while saving cost as the alternative of Dialysis is more expensive (38). More so, if KEPREPS may serve as a basis for reporting on model studies and the practice of emerging cross-national collaborations between KEPs (3) and future research expands it from a European standard to a global standard.

## Data Availability Statement

The original contributions presented in the study are included in the article/Supplementary Material, further inquiries can be directed to the corresponding author.

## Author Contributions

BM: literature review, coordinating expert panel work, and writing. MM: writing and synthesis of results. JK: literature review and writing. All authors contributed to the article and approved the submitted version.

## Conflict of Interest

The authors declare that the research was conducted in the absence of any commercial or financial relationships that could be construed as a potential conflict of interest.
