# Supplementary material for: Kidney Exchange Program Reporting Standards: Evidence-Based Consensus From Europe
Source: Front Public Health. 2021 Feb 11;9:623966. doi: 10.3389/fpubh.2021.623966 (PMC7928410; doi:10.3389/fpubh.2021.623966)
Supplement: Supplementary file 2 [file Data_Sheet_2.docx]

APPENDIX A

Expert panel

1. Lisa Burnapp: Clinical lead living donation at NHS Blood and Transplant, Consultant Nurse, Living Donor Kidney Transplantation at Guy’s & St. Thomas’ NHS Foundation Trust and Vice President British Transplant Society.
2. Peter Biro: Senior Research Fellow at CERS and Associate Professor at Corvinus University of Budapest, Optimization for Kidney Exchanges.
3. Paolo Ferrari: Founder of the Australian Kidney Exchange Program, Chief Medical Officer at Ente Ospedaliere Cantonale in Ticino, Academic Affiliation at the University of New South Wales and Università della Svizzeria Italiana.
4. Karine Hadaya: Nephrologist at Geneva University Hospitals.
5. Aline Hemke: Researcher at Dutch Transplant Society.
6. Christian Jaquelinet: Scientific counsellor, Head of the REIN registry and organ allocation simulation unit at Medical and Scientific Department, Agence de la biomédicine and Associated researcher, Paris-Saclay University.
7. Antonio Nicolò: Professor at Economics Department University of Padova and University of Manchester. Research into Kidney Exchange, including on Italy’s Kidney Exchange Protocol.
8. Matthew Robb: Senior Statistician at NHS Blood and Transplant.
9. Maria Valentin: Medical Officer at ONT (Spanish National Transplant Organization)
10. Ana Viana: Head of Research at INESC TEC; Professor at Polytechnic of Porto, Optimization for Kidney Exchanges

APPENDIX B

Table B1. List of expert’s reservations to the assigned measure category (column 'category'). The last six columns show the experts reservation towards the measure marked with 'X'

|  | Experts' reservations toward measures | | | | | | |
| --- | --- | --- | --- | --- | --- | --- | --- |
| **Measure name** | **assigned category** | **not-essential** | **not clear** | **living donor program-specific** | **difficult to measure** | **international KEP relevant** | **other** |
| Recipient & Donor Ethnicity | Extended Set |  |  |  |  |  | law constrains (France) |
| Recipient Gender | Base Set | X |  |  |  |  |  |
| Recipient & Donor Nationality | Extended Set |  |  |  |  | X | if the national program: no reporting, if international: Base Set |
| Recipient Prior Transplants | Base Set |  |  |  |  |  | more relevant at a sensitization |
| Computation Time | Extended Set | X |  |  |  |  |  |
| Number of Kidneys transplanted for which intended KEP recipient turned out infeasible | Extended Set | X | X |  |  |  |  |
| Donor LDKPI | Extended Set | X | X |  |  |  |  |
| Recipient & Donor Social Demographics | Extended Set | X | X |  |  |  |  |
| Cost Measures | Extended Set |  | X |  | X |  |  |
| Recipient/Donor/Graft survival | Base Set |  |  | X |  |  |  |
| Number of Rejection Episodes and Acute Rejections | Extended Set |  |  | X |  |  |  |
| QALY for Recipients/Donors | Extended Set |  |  | X | X |  |  |
| MFI - Threshold for (In)compatibility | Extended Set |  |  |  |  | X | different practices among HLA labs, |
| Criteria/definition of incompatibility | Base Set | X |  |  |  |  | Extended Set suggested |
| Acceptable Mismatch | Base Set | X |  |  |  |  | Extended Set suggested |
| Outcomes of alternate transplant programs | Extended Set |  | X |  |  |  | currently not reported by centers |
